# Testing the red reflex

**Published:** 2018-06-03

**Authors:** Richard Bowman, Allen Foster

**Affiliations:** 1 Honorary Clinical Consultant: International Centre for Eye Health, London School of Hygiene and Tropical Medicine, London, UK.; 2 Professor of International Eye Health; London School of Hygiene and Tropical Medicine, London, UK.

**A red reflex test can detect cataract and retinoblastoma. Both conditions require urgent referral.**

**Figure:**
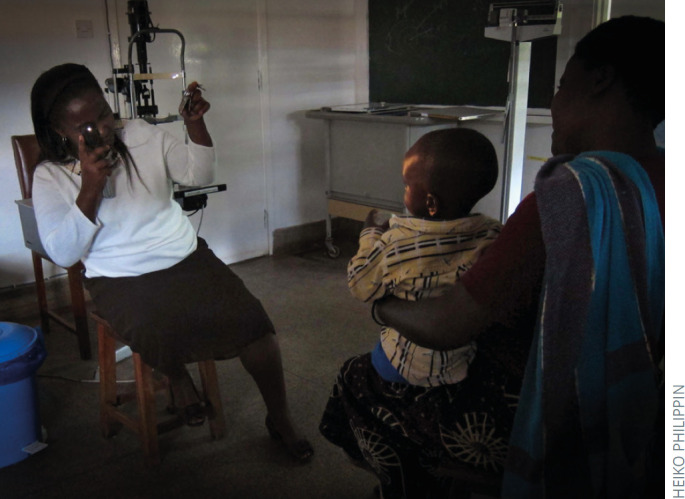
The red reflex is easier to see in a darkened room. TANZANIA

## Why is it important to test the red reflex?

The red reflex test can reveal problems in the cornea, the lens, the vitreous, and the retina; it is particularly useful in young children who may develop eye diseases but who are too young to complain of not seeing.

## What are the causes of an abnormal red reflex?

- Cataract
- Retinoblastoma
- Other uncommon diseases of the vitreous or retina

## When to test the red reflex for retinoblastoma

It is important to test the red reflex after birth, at the age of six weeks, during routine consultations, or when parents are concerned about the child's vision or the appearance of her or his eyes.

## How to test the red reflex

**Figure 1:**
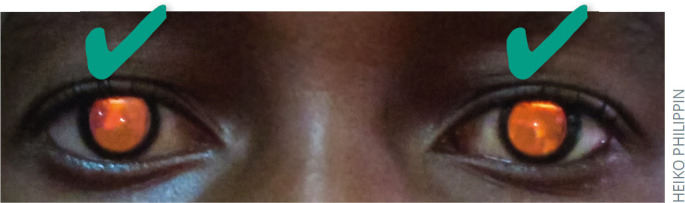
The normal red reflex

**Figure 2:**
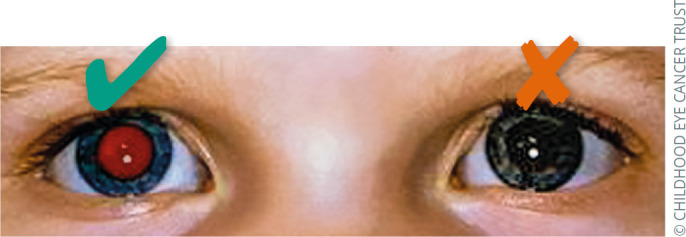
Right eye: the normal red reflex. Left eye: the absence of a red reflex is abnormal and could indicate a serious condition. Refer the child to a specialist.

**Figure 3:**
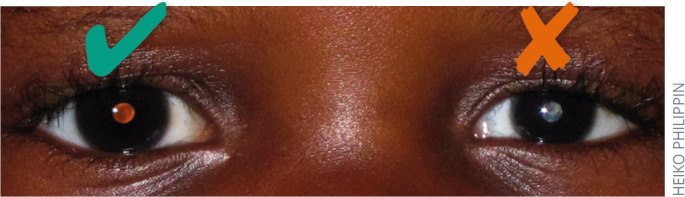
Right eye: the normal red reflex. Left eye: the wrong colour in a red reflex (here white) could indicate a serious condition. The child in this image has a cataract in the left eye. Refer the child to a specialist.

**Figure 4:**
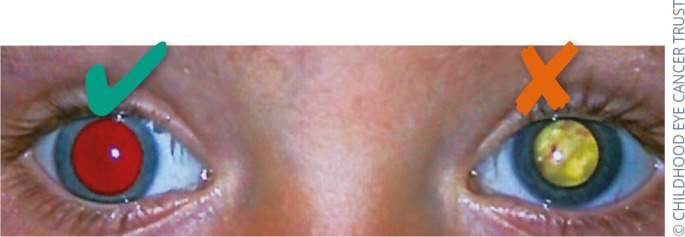
Right eye: the normal red reflex. Left eye: the wrong colour in a red reflex (here yellow-white) could indicate a serious condition. The child in this image may have retinoblastoma in the left eye. Refer the child to a specialist urgently.

- The red reflex is much easier to see in a darkened room, so switch off the lights and draw the curtains, or ask the parents and child to go with you into a room which is dark.
- Use a direct ophthalmoscope (or an ArcLight) with the lens power set at ‘0’. Make sure the batteries are charged.
- Sit about half a metre (50 cm) away. Hold the ophthalmoscope close to your eyes.
- Encourage the child to look at the light source and direct the light at the child's eyes. You should see an equal and bright red reflex from each pupil.
- Pay attention to the colour and brightness of the red reflex. It should be identical in both eyes (Figure 1). Any absence of the red reflex, or a difference between the eyes, or an abnormal colour in the pupil (Figures 2–4) may indicate retinoblastoma or another serious eye condition.

To determine whether the red reflex is normal, comparison with the red reflex of a parent may be helpful. If you are not sure whether the reflex is normal, dilate the pupil for a complete examination. **If you are unable to dilate the pupil, refer the child to a specialist.**

## What to do if the red reflex is abnormal

If possible, ask another colleague to check too. If the red reflex is abnormal, explain to the parents or carers that their baby/child may have an eye disease that will need to be treated. **Do not mention cancer or removal of the eye.**

Refer the child to a specialist for a complete eye examination. If possible, speak to the eye specialist by phone or text to explain the situation and confirm clinic times and dates.

Refer the baby/child to an eye specialist with an accompanying letter or note.

Make sure the parents know where to go and when. Emphasise that they must go in the next few days at the latest.

**Figure:**
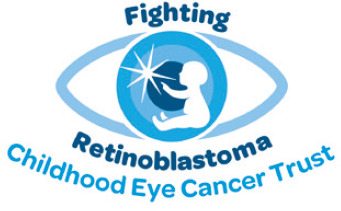

Adapted from the poster: ‘See RED’ produced by JR Ainsworth, UK National Retinoblastoma Service, Birmingham, UK and the Childhood Eye Cancer Trust. **www.chect.org.uk**. First published in the Community Eye Health Journal French edition, Issue 8, 2011.

**Figure 5:**
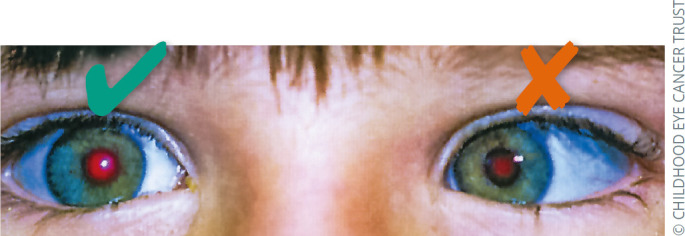
Right eye: the normal red reflex. Left eye: the red reflex is less bright and the corneal reflection (white spot on the cornea) is not centred. This is a squint, which may be the result of a serious underlying condition. Refer the child to a specialist.

